# Enactment of Third-Party Punishment by 4-Year-Olds

**DOI:** 10.3389/fpsyg.2012.00373

**Published:** 2012-10-01

**Authors:** Ben Kenward, Therese Östh

**Affiliations:** ^1^Department of Psychology, Uppsala UniversityUppsala, Sweden

**Keywords:** third-party punishment, moral development, norm violation, imitation, preschoolers

## Abstract

When prompted, preschoolers advocate punishment for moral transgressions against third parties, but little is known about whether and how they might act out such punishment. In this study, adult demonstrators enacted doll stories in which a perpetrator child doll made an unprovoked attack on a victim child doll, after which an adult doll punished either the perpetrator (consistent punishment) or victim (inconsistent punishment). When asked to help retell the story, given free choice of their own preferred actions for the adult doll, 4-year-olds (*N* = 32) were influenced by the demonstrated choice of target when selecting a target for punishment or admonishment. This influence was weak following inconsistent punishment, however, because the participants tended to change the story by punishing or admonishing the perpetrator when the demonstrator had punished the victim. Four-year-olds’ tendency to select a moral rule violator as a target for punishment is therefore stronger than their tendency to copy the specific actions of adults, which itself is known to be very strong. The evidence suggests that 4-year-olds’ enactment of punishment is at least partially based on a belief that antisocial actions deserve to be punished.

## Introduction

This study is an investigation of third-party punishment in preschool children. Third-party punishment refers to actions that inflict harm against an individual and that are motivated by that individual’s violation of social norms. Third-party punishment distinguishes itself from direct punishment in that third-party-punishment follows a norm violation that did not directly affect the punisher (Jensen, 2010). Direct punishment is known to occur in children (Robbins and Rochat, 2011) and non-human animals (Clutton-Brock and Parker, 1995) and is relatively straightforward to explain in functional and mechanistic terms, whereas third-party punishment is more challenging to explain and has so far only been identified in humans (Jensen, 2010). Third-party punishment (henceforth punishment) has a profound impact in that it enables the establishment of large scale cooperative networks by discouraging violations of cooperation norms (Boyd and Richerson, 1992; Fehr and Fischbacher, 2004; Nowak and Sigmund, 2005). The profound impact of punishment on society is also felt through the effects of modern criminal justice systems, which have been argued to be not entirely positive for society (Rubin, 2004; Useem and Piehl, 2008; The Economist, 2010).

It has been suggested on the basis of experiments (Price et al., 2002) and evolutionary models (Boyd and Richerson, 1992; Nowak and Sigmund, 2005; Gintis et al., 2008) that punitive sentiment may be an inherited specialized mechanism that evolved because individuals benefit from the cooperation that it enables (Robinson et al., 2007). Related to this is the finding that participants in experimental studies of attitudes toward punishment give justifications for punishment that are incompatible with the punishment judgments they actually advocate. Deterrence is frequently proposed as a justification for punishment, even though the majority are in fact motivated by a retributive sentiment that norm violations deserve punishment (Carlsmith and Darley, 2008; Keller et al., 2010). According to the evolutionary view, deterrence may be advocated because of rationalization, whereas retribution is practiced because that is how the evolved system works.

The profound impact of punishment on society and the suggestion that it may be an inherited trait mean that developmental studies of young children’s tendency to punish are essential. Despite this, the matter is almost completely unstudied. We are aware of only one published study in which young children have had the opportunity to inflict harm on those who harmed third parties (Hamlin et al., 2011; though see also Riedl et al., 2011). Hamlin et al. (2011) asked children in their second year (mean age 20 months) to give a treat to a puppet. The only source of treats were two other puppets who had already been given one treat each, one of whom had previous behaved antisocially and one of whom had behaved prosocially, and the participants were instructed to choose which puppet to take a treat from. They tended to take a treat from the antisocial puppet. Further, Hamlin et al. (2011) found that although infants normally avoid individuals who have behaved antisocially (Hamlin et al., 2007; Hamlin and Wynn, 2011), 8-month-olds actually preferred a puppet who had hindered rather than helped another puppet that had itself previously behaved antisocially. These results indicate that from very early in development, when forced to choose, children have a preference for causing harm to antisocial individuals and a preference for individuals that do likewise. These preferences appear to represent the developmental origins of the punitive sentiment.

Although there is a shortage of studies directly examining young children’s tendency to punish, there are many studies that have examined young children’s attitudes to social norm violations expressed in other ways (Smetana, 2006; Killen and Rutland, 2011). Children can verbally identify such violations by the age of three (Darley and Shultz, 1990; Catron and Masters, 1993; Tisak, 1993; Ingram and Bering, 2010). From this age children are also sensitive to the intention behind an action, separate from the outcome (Nelson, 1980; Zelazo et al., 1996; Nunez and Harris, 1998), and can also distinguish between violations of convention and morality (Smetana and Braeges, 1990; Stern and Peterson, 1999). Preschoolers’ behavior toward moral norm violators is altered appropriately – 3-year-olds avoid helping antisocial individuals (Vaish et al., 2010) and attempt to prevent antisocial acts (Vaish et al., 2011; Schmidt et al., 2012), and 4-year-olds prefer to allocate resources to prosocial rather than antisocial individuals (Kenward and Dahl, 2011). Gaze direction studies reveal that 3-month-olds are sensitive to distinctions between helping and hindering behavior (Hamlin et al., 2010). From the age of three children tend to answer yes when asked if norm violators should “get into trouble,” with moral norm violations regarded as more deserving of punishment that other social rules (Smetana, 1981, 2006; Smetana et al., 1993; Stern and Peterson, 1999).

To our knowledge, however, there are no published studies of young children’s attitudes toward norm violations in which children verbally administer a punishment, rather than advocating it in response to leading questions. Given the difference between study participants’ real and hypothetical moral behavior (FeldmanHall et al., 2012), and given that real punishment is a hostile and therefore risky act, a study in which young children were given the impression they had an opportunity to allocate real punishment would have the potential to greatly advance our understanding. That no such study has to our knowledge been published is striking given the above-mentioned reasons for the importance of understanding the development of punishment. The current authors submitted designs for two such studies to the Uppsala regional ethical review board, both of which were rejected.

The current study was designed to give preschoolers the opportunity to directly inflict verbally administered punishment. We used the technique of allowing children to guide the behavior of dolls because this was accepted by the Uppsala regional ethical review board, and because doll stories are a useful and valid alternative to observing real interaction in developmental research (Murray, 2007). Verbal punishment expression was investigated (in contrast to Hamlin et al., 2011) in order to be more revealing concerning children’s conceptual understanding of punishment. The primary hypothesis tested was that 4-year-olds target antisocial individuals when enacting punishment. The method used was to demonstrate stories involving verbal punishment of doll children by an adult doll following a violent attack by one of the children on the other, and then to engage the participant in retelling the story, encouraging them to change the story if they wanted. There were two within-subject conditions, one in which the adult doll punished the perpetrator of the attack (consistent punishment), and one in which the adult doll punished the victim of the attack (inconsistent punishment). The primary hypothesis predicts that participants will change the story in the inconsistent punishment condition so that the perpetrator rather than the victim is punished (see Welch-Ross and Schmidt, 1996 for an analogous method used to investigate the development of gender-stereotyping). Because children are such prodigious imitators, however, even of apparently inexplicable actions (Bandura et al., 1961; Horner and Whiten, 2005; Nielsen and Blank, 2011; Kenward, 2012; Over and Carpenter, 2012), it was also predicted that the demonstrated choice of punishment target would influence the participants’ actions. The demonstrated punishment was a withdrawal of privileges (sweets and TV). This punishment type was selected because it is not unusual in Sweden (Palmérus, 1999).

We also examined the participants’ attitudes to punishment by testing a secondary hypothesis that they would identify with and therefore want to play the role of a punisher, but only when the punishment was consistent. The idea behind this test was to be able to examine an actively and spontaneously expressed attitude toward punishment without explicit adult prompting or the need for participants to actually enact any punishment. After each story was demonstrated, children were asked to choose a doll to take the roll of in the retelling, selecting between the adult doll who had enacted the punishment and another adult doll who was similar except had not enacted any punishment. The hypothesis that 4-year-olds approve of punishment of antisocial individuals and therefore identify with those carrying out such punishment predicts that participants would choose to be the adult doll who had punished, but only when the punishment had been consistent. We also conducted an exploratory analysis of the effects of participants’ doll choice on their subsequent actions when retelling the story.

## Materials and Methods

### Participants

Participants were a self-selected sample who responded to an invitation letter sent to all families with children of appropriate age living in Uppsala, a medium-size Swedish city; therefore, participants were mostly ethnically Swedish and had mixed socioeconomic backgrounds. Included in the final sample were 32 4-year-olds (mean age 4 years and 1 month, SD = 1 month, 17 girls). Six additional participants were tested but excluded from the final analysis, four because of refusal to participate, one because of parental interference, and one because of experimenter error. One trial was excluded for each of three included participants due to experimenter error affecting only that trial.

### Materials

**Figure 1:**
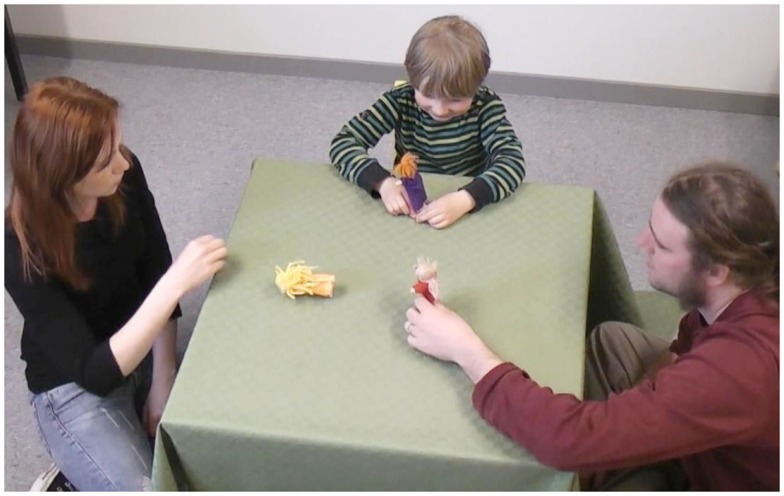
**Still from video recording of procedure**. Depicted is the moment during story retelling when one child doll has attacked the other, just before the participant is invited to select actions for the adult doll.

The procedure took place at a low table at which the participant sat on a chair, flanked by two experimenters sitting on the floor. A different set of four wooden dolls (two adults and two children), anatomically realistic and with clothes and hair, was used for each of four scenarios (Figure 1). In each scenario, each experimenter controlled one child doll and one adult doll (the same experimenter always had the same dolls in each scenario). Doll children were given normal Swedish names and their genders alternated from one scenario to the next, with boys used for the first scenario. The doll adults were nameless but in appearance matched the gender of the child dolls in their scenario.

### Design

Each participant took part in four trials, with each trial involving a different demonstrated story scenario, all of which followed the same pattern of one child doll attacking another, after which one child doll was punished by an adult doll. One experimenter always enacted the punishment with their adult doll and the other experimenter (known as the questioner) put questions to the child. The experimenter who enacted the attack with their child doll was varied over the four trials as AABB or BBAA. There were two trials for each participant for each of the two trial types (giving a within-subjects design): inconsistent punishment, in which the adult doll punished the victim child doll in the story demonstration, and consistent punishment, in which the adult doll punished the perpetrator child doll in the story demonstration. The order of inconsistent and consistent trials was varied as ABAB, BABA, ABBA, or BAAB, counterbalanced against the previous variable. The four different story scenarios (see Appendix) were always told in the same order.

### Procedure

The procedure was approved by the Uppsala Regional Ethics Committee. After welcoming the child and the parent to the study room, the two experimenters engaged the child in informal play intended to familiarize the child with the situation. They then explained the procedure to the parent and the child, and the parent signed an informed consent form. The four trials proceeded immediately one after another and lasted approximately 10 min in total.

Each trial began with a demonstration phase in which the experimenters told a scripted story (see Appendix) with the dolls. The child dolls were taken out and began by playing peacefully (football, hide-and-seek, rolling on the ground, and hand-standing, in the four scenarios in respective order of presentation), after which one of them, without provocation, insulted and attacked the other (by hitting, hitting, stamping on, and kicking, respectively). All aspects of the dolls’ actions in the demonstration were verbally narrated and acted out by the experimenters. For example, in the first scenario, the attack was depicted by an experimenter saying “Then [child doll’s name] said: you’re stupid, I’m going to hit you” and using the perpetrator child doll to knock over the victim child doll. After the attack, the two adult dolls were brought out and were declared to have seen everything that had happened. One adult doll (the non-punishing adult doll) was declared to do nothing. The other adult doll (the punisher adult doll), targeting the victim (inconsistent trials) or perpetrator (consistent trials) said “Why are you fighting, [child doll’s name]? Now you won’t get any sweeties. And you won’t get to watch TV either.” The punished doll did not react.

At this point, the choice phase was begun by the questioner saying “And that was the end of the story. [Participant’s name], if you got to be one of the grown-ups, which one would you be?” while pointing at both adult dolls simultaneously. The question was repeated after 10 s if necessary, and if after a further 10 s the participant had still not selected one of the adult dolls, they were handed the non-punishing doll. The non-chosen adult doll was removed.

At this point, the questioner began the retelling phase by saying “Now we are going to tell the story again, and [the other experimenter’s name] and I will be the children again, but [participant’s name], now you get to be the grown-up and decide what the grown-up does. It could be the same as before, or it could be something new. You decide.” The experimenters then performed the demonstration again, in a condensed form, stopping at the point at which the adults had previously intervened, at which point the questioner asked “what does the grown-up do now?” The question was repeated after 7 s if the participant did nothing. If the participant had still not caused the adult doll to take a clear action after an additional 7 s, the questioner asked “Does he/she do anything? Does he/she say anything”? If the participant still did nothing, the trial was over. If the participant did take any actions with the adult doll, the trial was over when the actions were completed. During the choice and retelling phases, both experimenters looked only at the participant, to avoid cuing actions with their gaze.

### Coding and analysis

All participants were coded from recordings by one of the experimenters and by a coder blind to the hypotheses of the study. The blind coder’s data was used in all analyses. Participants’ choices of adult doll were coded as being for the punisher, the non-punisher, or neither (Cohen’s κ = 1.0). Participants’ actions with the adult doll during story retelling were coded into the categories described in Table 1, with punishment actions also classified into subtypes (κ = 0.85). Actions sequences could contain actions classed in more than one category. Each action was also coded as targeting the victim, the perpetrator, or neither (κ = 0.94). Parametric model fit was checked by inspecting diagnostic scatterplots, using standardized residuals (Grafen and Hails, 2002).

**Table 1:** **Coding category definitions**.

Category	Observed actions
Unclear or irrelevant	Not interpretable (e.g., positioning the adult doll in front of a child doll with an unclear or absent verbalization) or irrelevant (e.g., declaring that the adult was picking flowers).
Prosocial	Causing a direct positive outcome for the target (e.g., declaring that the adult was comforting the child doll).
Admonishment	Reprimanding rather than punishing (e.g., a verbalization on the adult doll’s behalf commanding the child doll to apologize).
Punishment	Assignation of a negative outcome going beyond a verbal reprimand.
**PUNISHMENT SUBTYPES**	
Copied verbal punishment	Verbalization on the adult doll’s behalf assigning at least one of the same punishments as demonstrated (withdrawal of sweets or television).
Novel verbal punishment	Verbalization on the adult doll’s behalf assigning a punishment not used in the demonstration (e.g., “Now you have to go home and go to bed”).
Violent punishment	Using the adult doll to knock over the child doll.

## Results

**Figure 2:**
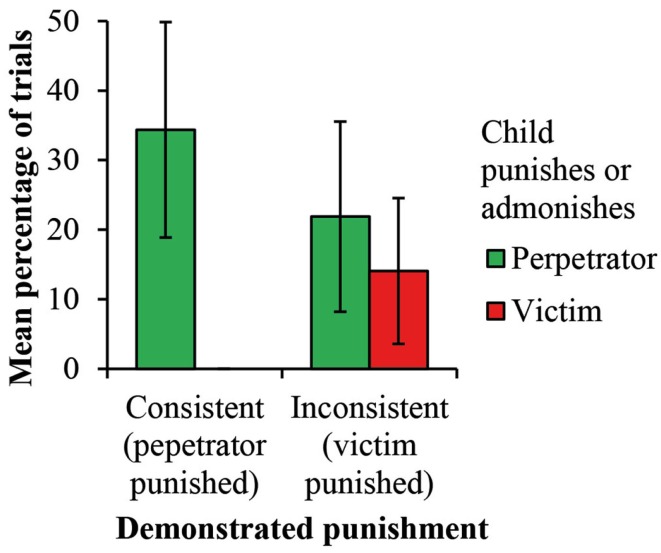
**Participants’ punishment and admonishment of the perpetrator and victim dolls following consistent and inconsistent punishment demonstrations**. Error bars show 95% confidence intervals of the individual means.

When participants caused the adult doll to punish or admonish, the trends in both conditions were for them to target the perpetrator more frequently than the victim (Table 2, Figure 2). This was confirmed by a 2 (condition: consistent vs. inconsistent demonstrated punishment) × 2 (participant target choice: perpetrator vs. victim) mixed-design ANOVA modeling the proportion of trials in which participants punished, which revealed a significant effect of participant target choice only, *F*(1, 93) = 12.77, *p* = 0.001, η^2^ = 0.08. The exact same pattern held when admonishment rather than punishment was modeled, target choice *F*(1, 93) = 6.30, *p* = 0.014, η^2^ = 0.04.

**Table 2:** **Mean percentage of trials in which participants caused the adult doll to perform certain actions**.

Participant’s actions	Consistent demonstration (perpetrator punished)			Inconsistent demonstration (victim punished)		
	Participant targets:			Participant targets:		
	Perpetrator	Victim	Neither	Perpetrator	Victim	Neither
**PRIMARY CATEGORIES**						
No action	0	0	41	0	0	48
Unclear or irrelevant action	8	8	8	5	9	2
Prosocial	0	3	0	0	5	0
Punish or admonish	34	0	2	22	14	2
**SUBCATEGORIES FOR PUNISHMENT AND ADMONISHMENT**						
Admonishment	16	0	0	12	8	2
Punishment (all types)	20	0	2	16	6	0
Novel verbal punishment	6	0	0	5	3	0
Copied verbal punishment	11	0	2	12	3	0
Violent punishment	8	0	0	3	2	0

*Participants targeted each child doll in turn in 5% of trials and targeted a child doll with more than one category of behavior in 7% of trials. This is why percentages for the primary categories sum to 104 and 107% for the consistent and inconsistent conditions, respectively, and also why totals for subcategories sum to more than parent category values*.

This does not, however, reflect a total lack of effect of demonstrated punishment target. Seven of 32 participants punished the victim at least once following an inconsistent punishment demonstration, but none did so following a consistent punishment demonstration, *p* = 0.016, sign test (Table 2, Figure 2). It also follows that participants were more likely to change the punishment target when retelling inconsistent punishment stories. This was confirmed by a 2 (condition: consistent vs. inconsistent demonstrated punishment) × 2 (participant target choice: same as demonstrated vs. different) mixed-design ANOVA modeling the proportion of trials in which participants punished, which revealed a significant interaction effect only, *F*(1, 93) = 12.77, *p* = 0.001, η^2^ = 0.08. The exact same pattern held when admonishment rather than punishment was modeled, interaction *F*(1, 93) = 6.30, *p* = 0.014, η^2^ = 0.04.

Participants caused the adult doll to punish or admonish in 36% of trials, and 50% of participants enacted punishment or admonishment at least once, with 44% targeting the perpetrator at least once, and 22% the victim. The proportion of trials containing punishment or admonishment did not differ between conditions, paired *t*(31) = 0.37, *p* = 0.712, *d* = 0.07 (Table 2). The proportion of trials in which participants performed no action was greater following inconsistent than consistent demonstrated punishment, paired *t*(31) = 2.4, *p* = 0.023, *d* = 0.42 (Table 2).

Participants made a choice of adult doll on 93% of trials. Of the choices made, 37% were for the punisher in consistent punishment trials and 40% were for the punisher in inconsistent punishment trials. The only pattern was therefore a general tendency for participants to avoid choosing the punisher: pooling the two conditions, the mean proportion of trials with a choice made in which the participant chose the punisher was significantly less than 0.5, *t*(31) = 2.15, *p* = 0.039, *d* = 0.38. A variety of exploratory analyses revealed no further effects on doll choice. For example, it was not affected by position in trial sequence.

After seven participants had been tested, the experimenters had a strong subjective impression that participants were choosing adult dolls based on appearance, because for example they answered “the red one” when asked to choose. At this point, the procedure was modified so that the questioner asked “Why did you choose that one?” after the participant had made a choice. Four of the next nine participants gave at least one answer consistent with a choice based on appearance, for example “because it’s red.” The procedure was then modified so that each pair of adult dolls was identical in appearance. Nevertheless, two of the next five participants gave at least one answer consistent with a choice based on appearance, for example “because it’s fatter” (the doll’s clothes had billowed out). At this point the extra question was abandoned as it had served its purpose and potentially placed additional demands on the participants. The change in doll appearance was made half-way through testing and was therefore counterbalanced with other variables. The presence of the question was approximately although not perfectly counterbalanced but had no detectable effect on other measured variables.

Finally the effects of participants’ doll choices on their subsequent actions when retelling the story were examined. When the perpetrator was punished in the demonstration (consistent trials), participants punished or admonished in 53% of trials in which they chose the punisher, but 30% of trials in which they chose the non-punishing adult doll. When the victim was punished in the demonstration (inconsistent trials), participants punished or admonished in 37% of trials in which they chose the punisher adult doll, but 43% of trials in which they chose the non-punishing adult. It therefore appeared that the tendency to punish or admonish after having chosen the punisher doll was greater when the demonstrated punishment was consistent. This was confirmed statistically by a significant interaction between condition (consistent vs. inconsistent) and participant’s choice of doll (punisher vs. non-punishing adult), *F*(1, 47) = 7.28, *p* = 0.010, η^2^ = 0.03, in a 2 × 2 mixed-design ANOVA modeling the proportion of trials containing punishment or admonishment in the retelling, including the aforementioned interaction and corresponding main effects.

## Discussion

The demonstration of a doll story in which the victim rather than the perpetrator of an antisocial act was punished (inconsistent punishment) caused some 4-year-olds to likewise enact punishment or admonishment for the victim when retelling the story. However, the trends after demonstrations of both consistent and inconsistent punishment were for participants to enact punishment for the perpetrator. This was because participants were more likely to change the story following an inconsistent punishment demonstration, so that the perpetrator rather than the victim was punished. Four-year-olds’ choices of targets for punishment or admonishment were therefore influenced weakly by the demonstration and strongly by a tendency to enact punishment for the perpetrator rather than the victim.

### Motivation for the imitation of inconsistent punishment

That some children should be caused by a demonstration to punish or admonish an innocent doll is no surprise. This result is to some extent a replication of classic studies demonstrating that preschoolers imitate both the general display of aggression and specific aggressive acts when adults display aggressive behavior toward dolls (Bandura et al., 1961; Bandura, 1965). A wealth of more recent studies demonstrate that preschoolers, older children, and even adults readily and faithfully imitate a range of different types of actions that don’t appear to make sense (McGuigan et al., 2011; Nielsen and Blank, 2011; Over and Carpenter, 2012). Children internalize adults’ strange actions as normative despite the absence of any explanation and although they are confused as to the actions’ purposes (Kenward et al., 2011; Kenward, 2012). The current results provide more evidence of young children’s faithful imitation, not only in that the demonstrated choice of punishment target was sometimes copied, but also in that most punishment actions enacted by participants included a verbal punishment copied directly from the demonstration.

### Motivation for the punishment of a moral norm violator

Against the background of these imitation results, the finding that participants usually changed the target when the demonstrated punishment was inconsistent is striking. It indicates that the tendency to prefer punishing antisocial rather than non-antisocial individuals is very strong in 4-year-olds, because it usually overcame the tendency to faithfully imitate, which is itself strong. This result is in line with findings showing that young children believe moral rules are to some extent authority independent (for example, they view hitting as wrong even in a hypothetical scenario in which an adult has condoned it, Smetana, 1985, 2006; Tisak and Turiel, 1988), but goes beyond these findings in showing that preschoolers also tend not to be guided by the morally inconsistent actions of a real and present adult.

This result is also consistent with previous studies in which preschoolers were asked to make recommendations concerning punishment in situations involving norm violations. From the age of 3 years children tend to answer yes when asked if norm violators should “get into trouble,” with moral norm violations regarded as more deserving of punishment than other social rules (Smetana, 1981, 2006; Smetana et al., 1993; Stern and Peterson, 1999). Here, however, children actually enacted punishment, albeit within the confines of a pretend doll world. Young children are aware of the pretend/reality distinction, but they generalize the workings of the real world to pretend worlds (Lillard, 2001). Importantly, children’s actions with dolls represent their real concerns and wishes, which is why doll stories are useful in research and clinical settings (Murray, 2007). The observed choices regarding punishment are therefore likely to reflect real behavioral tendencies. This study therefore represents an advance on previous studies in which children have only given opinions about what ought to happen, rather than acting themselves.

Although the participants’ choices of targets for punishment and admonishment were not usually influenced by the experimenters’ inconsistent punishment choices, it is likely that their target choices were influenced by their previous experiences of adult behavior. The specific styles of their punishment were clearly under such influence as they frequently involved behaviors which were not present in the demonstration but which had presumably been previously witnessed, such as instructions to go to bed early. Admonishment, which was never modeled, was almost as frequent as punishment. Also because the children were asked to play the role of an adult, it is therefore possible that their punishment and admonishment behavior was motivated primarily by a tendency to conform to the perceived desires of authority figures by emulating normal adult behavior (Damon, 1980; Kohlberg et al., 1983; Grusec and Kuczynski, 1997). A different possibility is that the participants were motivated (as adults are, Carlsmith and Darley, 2008; Keller et al., 2010) by an intrinsic desire to see punishment done to an individual who deserves it because the individual performed an antisocial act.

There are a number of reasons to believe that such an intrinsic deserts-based sentiment is at least partially responsible for motivating the participants’ punishment enactments. Firstly, if adult authority was solely responsible for determining participant behavior, one would expect currently present adults to have a stronger effect than they did (Nielsen and Blank, 2011). Secondly, even young preschoolers’ attitudes toward moral transgressions are (as previously discussed) to some extent independent of authority figures (Smetana, 1985, 2006; Tisak and Turiel, 1988). Thirdly, young preschoolers do have a sense of fairness, at least when it comes to what individuals deserve when resources rather than punishments are being allocated (Gummerum et al., 2010; Kenward and Dahl, 2011; LoBue et al., 2011). Together these findings indicate that the punitive behavior demonstrated here in some 4-year-olds is likely to build on an intrinsic desire for negative outcomes for those who have violated moral norms.

A second question concerns the origin of this punitive sentiment. The crucial role of internalization of cultural norms in the development of moral sentiment is clear (Thompson et al., 2006), but it is also possible that such attitudes may be to some extent biologically inherited. In non-verbal paradigms, even younger children than studied here demonstrate what appear to be the beginnings of punitive sentiments. Before their second birthday children prefer to cause a negative outcome to an antisocial individual, and 8-month-olds are attracted to an individual who hindered an individual that was antisocial (Hamlin et al., 2011). Given also observations that even infants have some expectations of fairness (Geraci and Surian, 2011; Schmidt and Sommerville, 2011; Sloane et al., 2012), the strong arguments that some aspects of moral behavior are biologically inherited (de Waal, 2008; Warneken and Tomasello, 2009), and the observation of continuity in social cognition from infancy to the preschool years in the context of antisocial behavior (Yamaguchi et al., 2009), it appears plausible that the development of punitive sentiment is partially determined by biological inheritance, but further work will be necessary to resolve this issue.

The finding that preschoolers modify a story which does not fit their normative conceptions parallels research carried out on the development of gender stereotypes. In such studies (e.g., Koblinsky et al., 1978; Welch-Ross and Schmidt, 1996) participants are told stories concerning children engaging in activities which are consistent or inconsistent with gender stereotypes. Participants’ memory for such stories after a delay is worse for stereotype inconsistent stories because, for example, they sometimes remember that a boy was playing with a truck when in the original story a girl had been doing so. The current study indicates that normative biases influence not only memory of behavior but also its re-enactment. However, because the current study did not include a delay and because inconsistent punishment is more unusual and salient than gender-stereotype inconsistent activities, the current effect is less likely to reflect a misremembering and more likely to reflect a modification of a correctly remembered story.

### Issues of validity

It should be noted that the frequency of punishment or admonishment was relatively low, with only slightly more than a third of trials containing punishment or admonishment, and only half of the participants ever enacting punishment or admonishment. This presents a challenge to the generalizability of the results and means that very universal conclusions should not be drawn on the basis of this study. It may be that not all 4-year-olds are motivated to enact punishment, even in a paradigm such as this which encourages it. However, it is also possible that children who did not enact punishment or admonishment may have avoided doing so for different reasons than a lack of motivation to punish. They may have felt uncomfortable in the situation and the demonstration of inconsistent punishment may have contributed to this. These possibilities are consistent with the observations that in many trials children took no action with the adult doll whatsoever, even apart from punishment or admonishment, and that this was more frequent following demonstration of inconsistent punishment.

### Lack of evidence for identification with punishers of moral norm violators

Our secondary hypothesis, that children would identify with a punishing agent when the punishment was consistent, and therefore choose to play the role of that agent when given the opportunity, was not supported. There was an overall weak tendency to choose the non-punishing adult, irrespective of whether the punishment was consistent. Many participants claimed to choose on the basis of doll appearance, and given the lack of an effect of condition on doll choice this suggests that dolls’ appearances were more important than dolls’ prior actions for determining which doll participants wanted to be. This makes sense when one considers that participants knew that they could choose new actions for the doll they selected but that they could not change its appearance. The lack of difference between the conditions and the readiness of the children to enact punishment indicate that the punisher was probably not avoided specifically because it punished. At the moment of choice, the punisher doll stood facing the targeted child doll, whereas the non-punishing adult doll faced the participant. This was intended to aid participants’ memory as to the dolls’ actions in the story, but given the participants’ unexpected responsiveness to doll appearance may have acted as a confounding factor.

Despite the lack of evidence that participants considered the actions of the adult dolls when choosing between them, doll choice did impact their subsequent actions with the doll. Participants were more likely to enact punishment or admonishment after choosing the punisher doll when the demonstrated punishment had been consistent than when it had been inconsistent. The simplest explanation for this result is that when retelling a story children have a tendency to cause a doll to perform the same actions as it previously performed, but that this tendency is diminished when the actions are incompatible with the children’s own preferences.

## Conclusion

The verbal nature of many of the punishment expressions showed that the children had a conceptual grasp of the appropriateness of the punishments they enacted. Children preferred to enact punishment toward those who had been antisocial, and this punishment was likely to have been at least partially motivated by a deserts-based sentiment that antisocial actions deserve to be punished. This is important because it suggests that the retributive tendencies which drive adult punishment (Carlsmith and Darley, 2008; Keller et al., 2010) are developing from an early age. It remains unclear, however, to what extent preschoolers would spontaneously punish if punishment had not been primed by a demonstration, or if a harmful action had not been required by the experimenter as in Hamlin et al.s’ (2011) study. There are still a very small number of published studies in which children have themselves had the opportunity to cause a negative outcome for other individuals who have behaved antisocially toward third parties (we are only aware of Hamlin et al., 2011). There is also still no study of which we aware in which young children have been given the impression they can allocate punishment to real people. If the associated ethical problems can be solved, such a study is the required next step in the investigation of the development of punitive sentiment.

## Conflict of Interest Statement

The author declares that the research was conducted in the absence of any commercial or financial relationships that could be construed as a potential conflict of interest.

## Appendix

### Procedure script, translated from Swedish

Two experimenters carried out the procedure, one of whom always controlled the punisher adult doll (P) and one of whom put all questions to the participant (Q). The experimenter who controlled the perpetrator child doll (E1) and the experimenter who controlled the victim (E2) were varied over the four trials as AABB or BBAA. The order of inconsistent and consistent trials was varied as ABAB, BABA, ABBA, or BAAB, counterbalanced against the previous variable. The four different story scenarios were always told in the following order.

**Table:**

Scenario 1	Scenario 2	Scenario 3	Scenario 4
***Demonstration phase***			
E1: This is [perpetrator child doll’s name, henceforth Perp]. He/she is four. *Takes out perpetrator child doll*.			
E2: And this is [victim child doll’s name, henceforth Vic]. He/she is four. *Takes out victim child doll*.			
E1: [Perp] and [Vic] were out playing one day.			
E2: [Vic] said, “shall we play football?”	E2: [Vic] said, “shall we play hide-and-seek?”	E2: [Vic] said, “shall we roll like sausages?”	E2: [Vic] said, “shall we do hand-stands?”
E1: “Yes let’s do that!”			
*The child dolls are made to play football with a small ball, borrowed from the participant*. E2: But then they got tired of football. E1: And then [Perp] said, “you’re stupid. I’m going to hit you!” *Causes Perp to knock over Vic*.	E2: [Vic] closed her eyes and started counting so [Perp] could hide. E1: But instead [Perp] said, “you’re stupid. I’m going to hit you!” *Causes Perp to knock over Vic*.	E2: They lay on the ground and started rolling around. *The child dolls are caused to do so*. E1: But then [Perp] jumped up and said, “you’re stupid. I’m going to stamp on you!” *Causes Perp to stamp on Vic, who stays down*.	E2: [Vic] did a hand-stand. *Causes Vic to do so*. E1: But [Perp] said “you’re stupid. I’m going to kick you in the head!” *Causes Perp to kick Vic, knocking Vic over*.
E2: And she/he hit/stamped on/kicked [Vic], even though [Vic] hadn’t done anything. “Ouch,” said [Vic].			
Q: There was a grown-up who had seen everything. *Takes out non-punishing adult doll*.			
P: There was another grown-up who had also seen everything. *Takes out punisher adult doll*.			
Q: The first grown-up didn’t say anything.			
P: *Positions adult doll facing Perp (consistent)/Vic (inconsistent)*. But the other grown-up said to [Perp/Vic],			
“Why are you fighting, [Perp/Vic]? Now you won’t get any sweeties. And you won’t get to watch TV either.”			
***Choice phase***			
Q: And that was the end of the story. [Participant’s name], if you got to be one of the grown-ups, which one			
would you be? *Points at both adult dolls simultaneously*.			
*Further details of the choice procedure including further prompting are found in the method*.			
***Retelling phase***			
Q: Now we are going to tell the story again, and [P’s name] and I will be the children again, but [participant’s name], now you get to be the grown-up and decide what the grown-up does. It could be the same as before, or it could be something new. You decide.			
E2: [Perp] and [Vic] were out playing football (*the child dolls are briefly caused to do so*), but then they got tired of it. E1: And then [Perp] said, “you’re stupid. I’m going to hit you!” *Causes Perp to knock over Vic*.	E2: [Perp] and [Vic] were out playing hide-and-seek. [Vic] closed her eyes and started counting so [Perp] could hide. E1: But instead [Perp] said, “you’re stupid. I’m going to hit you!” *Causes Perp to knock over Vic*.	E2: [Perp] and [Vic] were out rolling like sausages. *The child dolls are caused to do so*. E1: But then [Perp] jumped up and said, “you’re stupid. I’m going to stamp on you!” *Causes Perp to stamp on Vic*.	E2: [Perp] and [Vic] were outdoing hand-stands. *Causes Vic to do so*. E1: But [Perp] said “you’re stupid. I’m going to kick you in the head!” *Causes Perp to kick Vic, knocking Vic over*.
E2: And she/he hit/stamped on/kicked [Vic], even though [Vic] hadn’t done anything. “Ouch,” said [Vic].			
Q: And what does the grown-up do now?			
*Further details of the retelling procedure including further prompting are found in the method*.			
